# Changes in body mass index and its association with socio-demographic characteristics between 2010 and 2016 in Singapore

**DOI:** 10.3389/fpubh.2024.1374806

**Published:** 2024-03-27

**Authors:** Yunjue Zhang, Edimansyah Abdin, Rajeswari Sambasivam, Saleha Shafie, Kumarasan Roystonn, Janhavi Ajit Vaingankar, Siow Ann Chong, Mythily Subramaniam

**Affiliations:** Research Division, Institute of Mental Health, Singapore, Singapore

**Keywords:** epidemiology, body mass index, obesity, obese, overweight

## Abstract

**Background:**

Epidemiological studies have observed an increase in the prevalence of obesity in both western and Asian countries. This study aims to compare the distribution of body mass index (BMI) in the general population of Singapore between 2010 and 2016, and to explore the socio-demographic risk factors associated with it.

**Methods:**

Data for this study were extracted from two national-wise studies in 2010 and 2016, two population-based, cross-sectional epidemiological studies. BMI cut-off scores were used as an indicator to assess obesity in this study, and the data included in the analysis was self-reported by the respondents.

**Results:**

Overall, the study observed decreasing prevalence in underweight and normal weight categories; and an increasing prevalence in overweight and obesity categories in the Singapore adult population between 2010 and 2016. Age, gender, ethnicity, marital status, and educational level were found to be significantly associated with BMI categories.

**Conclusion:**

The observed increase in the population’s BMI between 2010 and 2016 may lead to an increase in the incidence of chronic diseases in Singapore. Our study findings add to the existing local literature and provides data for evidence-based policymaking on health-related interventions and program planning.

## 1 Introduction

Overweight and obesity are known to be important contributors to adverse health consequences (1, 2). At an individual level, they are associated with various physical diseases, including cardiovascular diseases (3), several types of cancers, and diabetes mellitus (4). Consequently, at the population level, having excessive body weight not only affects an individual’s physical health but could also lead to poor mental health (5), lowers one’s quality of life and reduces life expectancy (6). At the population level, overweight and obesity are a public health concern, resulting in excessive health care costs to society.

The Global Burden of Disease Study Report (7) showed that the global prevalence of overweight increased from 26.5 to 39.0% from 1980 to 2015. The global prevalence of obesity likewise rose from 7 to 12.5% from 1980 to 2015, representing an estimated increase of 50% in the global prevalence of overweight and 80% in the global prevalence of obesity, with the American and European regions having the highest prevalence. In North America, the prevalence of overweight increased from 45.3 to 64.2% from 1980 to 2015, and the prevalence of obesity increased from 12.9 to 28.3% from 1980 to 2015. While in South East Asia, Wang et al. and Chooi et al. reported the prevalence of overweight increased from 10.9 to 24.3% from 1980 to 2015, and the prevalence of obesity increased from 1.7 to 6.2% from 1980 to 2015 (8, 9).

In relation to gender and socioeconomic status, the same report (9) found that the prevalence of overweight and obesity is higher in females than in males and this gender difference remained persistent across time. Obesity is also more common among middle-aged adults, especially wealthy females in low-income countries, while obesity affects both genders among the disadvantaged groups, those who experience a higher risk of poverty, social exclusion, discrimination, and violence in high-income countries (10).

However, Asians generally have a higher percentage of body fat than Caucasians of the same gender and age, and the risk of developing type 2 diabetes and cardiovascular diseases is higher among Asians than among Caucasians with the same body mass index (BMI) score (cut-off score at 25) (11). Thus, the current global cut-off points do not provide an adequate basis for many populations in Asia in relation to understanding or giving recommendations for those in the overweight and obese category. Thus, studies have suggested different categories for Asians which are at lower cut-offs (12).

Singapore has a unique multi-ethnic composition, comprising Chinese (74.3%), Malays (13.4%), Indians (9.0%), and other ethnic groups (3.3%) (13). With evidence suggesting a different relationship between ethnic groups and body fat percentage (14, 15). The Singapore Health Promotion Board-Ministry of Health Clinical Practice Guidelines indicate significant variations in obesity prevalence among different ethnic groups within Singapore, with Malays having the highest prevalence of obesity at 20.7%, followed by Indians at 14.0%, and Chinese at 5.9% (12). Another cross-sectional study conducted in Singapore found significant ethnic variations in body fat distribution, with Indian females showing the highest body fat percentage (38.2%) and Chinese males the lowest among the groups studied (24.4%) (16). These differences in obesity rates among ethnic groups may be attributed to a combination of various factors, namely genetic, cultural, dietary, and lifestyle (17). A higher proportion of body fat, regardless of body weight or BMI, can significantly increase various health issues, including cardiovascular diseases and diabetes (18). Given the ethnic differences, it is important to examine the changes in BMI across time in each ethnic group as it could have public health implications.

The current study aims to compare the distribution of prevalence of various BMI categories using Asian BMI cut-offs between 2010 and 2016 using self-reported height and weight measurements using data from the Singapore Mental Health Study 2010 (SMHS 2010) and Singapore Mental Health Study 2016 (SMHS 2016) (19, 20) to explore the changes and the socio-demographic risk factors in the Singapore adult population.

## 2 Materials and methods

### 2.1 Respondents and procedures

Both SMHS 2010 and SMHS 2016 were conducted using the same procedures (20, 21). Both studies were cross-sectional, population-based epidemiological studies conducted among Singaporeans and Permanent residents aged 18 years and above living in Singapore. The sample was drawn from an administrative database of all citizens and permanent residents in Singapore and updated regularly.

An invitation letter was sent to each randomly selected participant/resident, followed by a personal home visit by a trained interviewer from the designated survey company to obtain their agreement to participate in the survey. Once participants agree, a trained interviewer will conduct face-to-face interviews at participants’ preferred time and venue. The survey was available in English, Chinese, and Malay languages, and each participant was asked to select the language in which they were comfortable speaking before commencing any study procedures. Residents who were having severe physical or mental conditions, were living in institutions or hospitals, were not able to speak the above-mentioned languages, or living overseas at the point of the survey, and those who were not contactable due to incomplete or incorrect addresses, were considered ineligible cases and were excluded from the study.

Ethics approval for this study was obtained from the National Healthcare Group Domain Specific Review Board (DSRB No.: 2015/01035). Written informed consent was obtained from all participants prior to the survey, participants could also choose their preferred language (English, Mandarin, or Malay) for the consent as well as the survey. Parental consent was also obtained for minors, i.e., those aged 18–20 years. All study procedures were performed in accordance with DSRB’s ethical guidelines and regulations.

### 2.2 Data collection

#### 2.2.1 Anthropometric measurements

Self-reported height and weight were collected from respondents as part of the data collected in the sociodemographic questionnaire.

#### 2.2.2 Sociodemographic information

Data on gender (female, male), age, ethnicity (Chinese, Malay, Indian, and Others), marital status (single, married, divorced/ separated, or widowed), educational level (primary and below, secondary, vocational institute, pre-university/ junior college, diploma, and university), employment status (employed, unemployed and economically inactive) was collected.

#### 2.2.3 Body mass index

BMI is defined as the weight in kilograms divided by the square of the height in meters (BMI = body mass/(height)^2^). The BMI classification scores were categorized according to World Health Organization (WHO) guidelines. Those having BMI scores of 30 and above were classified as obese, those with BMI of 25–29.9 were defined as overweight, 18.5–24.9 were considered to be in the normal range, and BMI below 18.5 was classified as underweight (WHO, 2000).

### 2.3 Statistical analysis

All estimates were weighted to ensure the results represented Singapore’s general population. To examine the associations between BMI categories and socio-demographic variables, chi-square (χ^2^) tests were used, followed by multinomial logistic regressions. A significant change in BMI categories between two surveys in sociodemographic groups was further tested in the pooled multinomial logistic regression analyses by adding interaction terms between the year of the study and each demographic variable with adjustment of sociodemographic factors. Year of survey (SMHS-2010 = 0, SMHS-2016 = 1), age groups, gender, ethnicity, marital status, education, and employment status were predictors in the regression model. Statistical significance was evaluated at the <0.05 level using two-sided tests. All statistical analyses were performed using the Statistical Analysis Software (SAS) System version 9 (SAS Institute Inc., 2011).

The non-response rates for SMHS 2010 and 2016 were 24.1 and 31.0%, respectively (20, 21).

## 3 Results

Table 1 summarizes the prevalence in 2010 and 2016 according to BMI categories. Table 2 presents the changes in BMI categories between 2010 and 2016 and the association between each category and socio-demographic characteristics.

**Table 1:** Sociodemographic characteristics and body mass index (BMI) categories in 2010 and 2016.

Overall sample		2010									2016								
		Body Mass Index Categories																	
		Underweight		Normal Weight		Overweight		Obesity			Underweight		Normal Weight		Overweight		Obesity		
		*(<18.5 kg/m^2^)*		*(= > 18.5 < 25 kg/m^2^)*		*(= > 25 < 30 kg/m^2^)*		*(= > 30 kg/m^2^)*			*(<18.5 kg/m^2^)*		*(= > 18.5 < 25 kg/m^2^)*		*(= > 25 < 30 kg/m^2^)*		*(= > 30 kg/m^2^)*		
		n	%	n	%	n	%	n	%	*p* value	n	%	n	%	n	%	n	%	*p* value
Total		446	8.4	2,141	39.9	2,231	35.1	1,473	16.7		289	6.7	1,581	36.5	2061	38.6	1,454	18.2	
Sociodemographic																			
Age group	18–34	273	13.3	950	46	646	26.6	395	14.1	<0.001	135	8.9	640	46.5	528	30.5	354	14.2	<0.001
	35–49	97	6.4	705	39.3	913	37	593	17.3		58	6.2	346	31.5	566	41	457	21.2	
	50–64	60	5.6	385	34.4	537	39.3	419	20.7		49	4.6	347	32.2	572	41.6	437	21.5	
	65 and over	16	5.9	101	32.2	135	46	66	13.9		47	6.5	248	32.3	403	47.7	206	13.5	
Gender	Male	146	4.4	968	34	1,294	42	783	19.8	<0.001	111	4.5	713	30.1	1,225	45.4	737	20	<0.001
	Female	300	12.1	1,173	45.5	937	28.6	690	13.7		178	8.8	868	43	836	31.8	717	16.4	
Ethnicity	Chinese	185	9	849	42.4	669	34.8	273	13.8	<0.001	119	7.3	655	40	646	39.1	218	13.6	<0.001
	Malay	144	6.7	668	31.2	684	32	649	30		76	4.9	359	23	565	34.6	606	37.6	
	Indian	108	5.9	534	28.7	766	39.9	497	25.4		72	4.4	408	24.6	656	38.6	538	32.5	
	Others	9	4.2	90	36.1	112	41.5	54	18.1		22	4.8	159	34	194	41	92	20.2	
Marital status	Never married	241	13.5	770	44.5	488	27.8	288	14.1	<0.001	138	9.7	584	44.7	457	32	291	13.6	<0.001
	Married	175	6	1,222	38	1,588	38.1	1,091	17.8		113	4.9	849	32.6	1,402	41.6	1,028	20.9	
	Divorced/separated	19	9.3	93	42.9	89	31.5	47	16.4		21	8.57	98	34.2	101	38	75	19.2	
	Widowed	11	6.4	56	20.9	64	44.8	47	18		17	6.8	58	29.4	101	51.6	60	12.3	
Education	Primary and below	12	6.1	66	31.9	69	42.3	56	19.7	<0.001	49	8.4	188	28.3	299	41.8	211	21.6	<0.001
	Secondary	48	6.4	227	34.1	293	39.5	245	20		68	5.2	365	32.8	557	40.7	461	21.4	
	Pre-Uni./	123	8.2	572	37.6	670	35.3	540	18.9		20	8.7	108	45.3	106	35.7	61	10.3	
	Junior College																		
	Diploma	65	10.9	236	35	222	31.9	183	22.3		46	5.9	333	40.2	349	35.9	269	18	
	Vocational	113	8.9	535	45.3	435	30.4	248	15.3		33	5.14	109	25.8	174	42.5	162	26.6	
	University	85	9.1	505	44	542	36.3	201	10.7		73	7.4	478	40.7	576	37.3	290	14.5	
Employment	Employed	304	8.1	1,487	38.8	1,630	35.9	1,043	17.2	0.2	170	5.6	1,091	36.1	1,457	39.3	1,028	19	0.02
	Economically inactive	103	9	498	43.1	434	33.1	330	14.8		95	9.2	406	38	502	36.9	353	15.9	
	Unemployed	30	11.9	100	40.7	93	29.7	62	17.8		24	10.4	84	35.6	101	36.9	73	17.1	

**Table 2:** Pooled multinomial logistic regression models for changes in BMI categories* between 2010 and 2016 periods (Interaction**).

Sociodemographic factors		Underweight at 2016				Overweight at 2016				Obesity at 2016			
Interaction terms with year of survey		OR	95% CI		*p* value	OR	95% CI		*p* value	OR	95% CI		*p* value
Age group	18–34 (2010)	Ref				Ref				Ref			
	35–49 (2016)	1.8	1.0	3.2	0.1	1.1	0.8	1.6	0.5	1.4	1.0	2.1	0.9
	50–64 (2016)	1.2	0.6	2.4	0.6	0.9	0.6	1.2	0.4	0.8	0.5	1.3	0.4
	65 and over (2016)	1.6	0.6	3.9	0.3	0.9	0.5	1.5	0.6	0.8	0.4	1.7	0.7
Gender	Male (2010)	Ref				Ref				Ref			
	Female (2016)	0.7	0.4	1.1	0.1	1.0	0.7	1.2	0.7	1.0	0.8	1.3	0.7
Ethnicity	Chinese (2010)	Ref				Ref				Ref			
	Malay (2016)	1.2	0.7	1.8	0.5	1.2	0.9	1.5	0.2	1.4	1.1	2.0	0.02
	Indian (2016)	1.0	0.7	1.5	1.0	0.9	0.7	1.2	0.6	1.4	1.0	1.9	0.03
	Others (2016)	1.4	0.6	3.5	0.5	1.0	0.7	1.5	1.0	1.3	0.8	2.2	0.3
Marital status	Never married (2010)	Ref				Ref				Ref			
	Married (2016)	1.2	0.8	2.0	0.4	1.0	0.7	1.3	0.7	1.2	0.8	1.7	0.4
	Divorced/separated (2016)	1.6	0.6	4.4	0.4	1.1	0.6	2.1	0.8	1.1	0.5	2.5	0.8
	Widowed (2016)	1.3	0.3	5.0	0.7	1.0	0.4	2.0	0.9	0.6	0.2	1.7	0.3
Education	University (2010)	Ref				Ref				Ref			
	Primary and below (2016)	2.0	0.7	5.8	0.2	1.0	0.5	1.9	1.0	0.7	0.3	1.4	0.3
	Secondary (2016)	0.9	0.5	1.9	0.9	1.0	0.7	1.6	0.7	0.7	0.4	1.2	0.2
	Pre-Uni./Junior College (2016)	0.8	0.4	1.9	0.7	1.0	0.6	1.5	0.9	0.4	0.2	0.8	0.01
	Diploma (2016)	0.5	0.3	1.0	0.07	1.2	0.8	1.8	0.4	0.7	0.4	1.2	0.2
	Vocational (2016)	1.2	0.6	2.6	0.76	1.8	1.1	2.9	0.02	1.2	0.7	2.2	0.5
Employment	Economically inactive (2010)	Ref				Ref				Ref			
	Employed (2016)	0.7	0.4	1.1	0.1	0.9	0.7	1.2	0.5	0.9	0.6	1.4	0.8
	Unemployed (2016)	0.8	0.3	2.1	0.7	1.1	0.6	2.1	0.8	0.8	0.4	1.9	0.7

*BMI reference, normal weight range. **Odds ratio were estimated using a series of pool multinomial logistic regression by adding interaction terms between each sociodemographic variable and year of survey.

### 3.1 Overall prevalence

Overall, 6.7% were underweight in 2016 vs. 8.4% in 2010, 36.5% were in the normal range in 2016 vs. 39.9% in 2010, 38.6% were overweight in 2016 vs. 35.1% in 2010, while 18.2% were obese in 2016 vs. 16.7% in 2010.

### 3.2 BMI and associated factors across the two surveys

#### 3.2.1 Age groups

BMI was significantly associated with age in both 2010 and 2016 (*p* < 0.001; Table 1). However, no statistically significant associations were observed between BMI categories and age across the two cohorts (Table 2).

#### 3.2.2 Gender

BMI was significantly associated with gender in both 2010 and 2016 (*p* < 0.001; Table 1). However, no statistically significant associations were observed between BMI categories and gender across the two cohorts (Table 2).

#### 3.2.3 Ethnicity

BMI was significantly associated with ethnicity in both 2010 and 2016 (*p* < 0.001; Table 1). Compared to Chinese ethnicity, those of Malay ethnicity were 1.4 times more likely to be in the obese category in 2016 than in 2010 (*p* = 0.02; OR = 1.4, 95% CI:1.1, 2.0; Table 2). In addition, those of Indian ethnicity were 1.4 times more likely to be in the obese category in 2016 than in 2010 (*p* = 0.03; OR = 1.4, 95% CI:1.04, 1.9; Table 2).

#### 3.2.4 Marital status

BMI was significantly associated with marital status in both 2010 and 2016 (*p* < 0.001; *p* < 0.001; Table 1). However, no statistically significant associations were observed between BMI categories and marital status across the two cohorts.

#### 3.2.5 Education level

BMI was significantly associated with education level in both 2010 and 2016 (*p* < 0.001; Table 1). As compared to respondents with a university degree, respondents with vocational education were 1.8 times more likely to be overweight in 2016 than in 2010 (*p* = 0.02; OR = 1.8, 95% CI:1.1, 2.9; Table 2); for those with pre-university/junior college certifications, they were 0.4 times less likely to become obese in 2016 than in 2010 (*p* = 0.01; OR = 0.4, 95% CI:0.2, 0.8; Table 2).

## 4 Discussion

Our findings showed an overall decrease in the underweight category (from 8.4% in 2010 to 6.7% in 2016) and in the normal weight category (from 39.9% in 2010 to 36.5% in 2016) and an increase in the overweight category (from 35.1% in 2010 to 38.6% in 2016) and obese category (from 16.7% in 2010 to 18.7% in 2016) in the Singapore adult population, which is consistent with the global trend that has been observed in other studies. In 2008, about 1·5 billion adults globally were overweight, and 502 million adults were obese. While the epidemic started in high income-countries later, a similar increase was seen in middle and low-income countries (mostly in high socioeconomic countries) (10). In 2014, 39% of adults were overweight. 11% of men and 15% of women worldwide were obese. In the South East Asia region, the prevalence of overweight and obese were 22 and 5%, respectively (6).

From a systemic perspective, rising socio-economic status affects the population’s lifestyle. People tend to be more sedentary due to longer working hours and are less able to devote time to vigorous physical activities, especially as jobs have become more office-based (22). Furthermore, the convenience of transportation also plays a part in encouraging sedentary behavior (10). A study conducted in China found that obesity prevalence is likely to increase among families that own auto-motor vehicles (23). In Singapore, the car population, has increased from 2010 to 2016 (24) suggests that this could be another contributing factor.

Increased calorie content of food has been used to explain the global rise in body weight and obesity (25). Overconsumption of food is promoted in the form of increased portion size and more affordable fast food that are high in energy but poor in nutrition (26). Unhealthy food is more accessible than healthy and nutritious food in the market (27). Singapore’s active night-life culture, including late-night suppers, might also be associated with the current increasing trend (28). Emerging literature suggests feeding times may have an effect on metabolism rate and hence influence the development of obesity (29, 30).

The current study found that age was significantly associated with BMI in both 2010 and 2016. With the economic growth in Singapore in the past couple of decades, people have more access to more nutrition-dense food resulting in changes in their health status (31). Many studies suggest that the direct reason being human basal metabolism decreases with age due to muscle loss and an increase in body fat (32, 33).

Gender was also found to be significantly associated with BMI in both 2010 and 2016. This finding is in line with other studies conducted on differences in body mass index and body weight perceptions between the two genders (34, 35). One explanation could be that females generally have a higher body fat composition than males (36). A locally based population study found that females, compared to males, are less likely to exercise regularly (22). Few other studies also found that young females are more sedentary than males because they enjoy socializing more, such as sitting and talking, which could be another possible explanation for current findings on the gender difference (37, 38).

Compared to Chinese ethnicity, Malay ethnicity and Indian ethnicity were found to be more likely in the obese category in 2016 than in 2010. This finding is in line with a local study where they found Malay ethnic group and Indian ethnic group have higher BMI than the Chinese ethnic group. This could be due to different dietary practices among different ethnic groups. For instance, Malays also were shown to consume more of “confectionery and sweeteners” food groups relative to Chinese and Indians (24). However, there are more recent studies have shown the contrary where Chinese ethnic groups are reported to consume more western fast food than the other two ethnic groups. Additionally, more longitudinal study findings are needed to explain why within the same ethnic group, people are more likely to be in the obese category in 2016 than in 2010. One of the possible reason could be the increase of consumption of processed food.

Compared to degree holders, diploma holders were found less likely to be underweight in 2016 than in 2010, while those with vocational education were more likely to be overweight in 2016 than in 2010. Study findings also suggested that students with pre-university/junior college qualifications are less likely to become obese in 2016 than in 2010. While the findings are consistent with existing literature on the significant association between educational levels and obesity-related behavior (39), Results did not show a significant inverse relationship between the education levels and BMI categories. Changes between the two time periods for individuals with the same educational level might be explained due to the positive effects of ongoing national programs in local schools; for example, healthy eating guidelines are provided to help students choose healthy food and beverages (12). Information regarding healthy ingredients and their suppliers are complied on an official website for school canteen vendors’ reference (40).

### 4.1 Limitations

Data were collected through self-report from the respondents, so the measurement was subjective, the weight could be under-reported due to embarrassment, and both height and weight could be misreported due to inaccurate recollection. However, the mean differences between self-reported and measured anthropometric values were insignificant in this population (39). Thus, the use of self-reported anthropometric values should not affect the validity of the conclusions drawn in this study. Data did not include those below 18 years of age, which may be an important group to explore.

To mitigate these limitations in future studies, strategies should be implemented to minimize reporting bias. This can be done by stressing the importance of objective measures of weight and height, and interviewers presenting themselves professionally which may reduce the embarrassment felt by participants who are in the overweight or obese BMI categories. Future studies could consider longitudinal design which can track changes over time, in the same cohort which can help avoid some of the confounders. Further research is needed to identify which indicators or combination of indicators would provide the best estimation of excess body fat in population surveys (41).

## 5 Conclusion

Obesity has become a serious public health concern globally and locally, resulting in multiple chronic diseases affecting an individual’s quality of life. Hence, the increasing prevalence of obesity in Singapore is burdening the healthcare system, costing the nation approximately 260 million per year on disease-related treatments and comorbidities (42). In 2016, obesity-related expenditure rose to at least 400 million (12). To alleviate the burden, several agencies have ramped up their efforts and rolled out ongoing national campaigns to promote an active lifestyle among the local residents (43, 44). However, more needs to be done, such that agencies should develop programs that are culturally sensitive for each ethnic as well as educational group.

## Data availability statement

The original contributions presented in the study are included in the article/supplementary material, further inquiries can be directed to the corresponding author.

## Ethics statement

The studies involving humans were approved by the National Healthcare Group Domain Specific Review Board (DSRB No.: 2015/01035). The studies were conducted in accordance with the local legislation and institutional requirements. The participants provided their written informed consent to participate in this study.

## Author contributions

YZ: Conceptualization, Investigation, Methodology, Writing – original draft, Writing – review & editing. EA: Data Curation, Formal Analysis, Writing – review & editing. RS: Writing – review & editing. SS: Writing – review & editing. KR: Writing – review & editing. JV: Funding Acquisition, Writing – review & editing. SC: Funding Acquisition, Writing – review & editing. MS: Conceptualization, Funding Acquisition, Supervision, Writing – review & editing.
